# Suggestive

**Published:** 1884-04

**Authors:** 


### SUGGESTIVE.

Many good friends of this journal have written complimentary letters to the editor and publishers. Some have announced a readiness to help us pecuniarily if it were needed. They think the *I. P.* has a mission, and should be sustained, and they are willing to liberally contribute for this purpose. We deeply appreciate all these kind words, and may we not deferentially hint to such that there is a way in which they can assist us, and at the same time perform an act of kindness to their neighbors. It is by soliciting subscriptions. If every friend of the *I. P.* would secure one new subscriber, he would practically exhibit his sympathy, and enable us to make it more nearly our ideal of what a dental journal should be. Remember that every dollar paid to it goes into the journal.

